# Metaproteomics Reveals Similar Vertical Distribution of Microbial Transport Proteins in Particulate Organic Matter Throughout the Water Column in the Northwest Pacific Ocean

**DOI:** 10.3389/fmicb.2021.629802

**Published:** 2021-03-25

**Authors:** Ling-Fen Kong, Ke-Qiang Yan, Zhang-Xian Xie, Yan-Bin He, Lin Lin, Hong-Kai Xu, Si-Qi Liu, Da-Zhi Wang

**Affiliations:** ^1^State Key Laboratory of Marine Environmental Science, College of the Environment and Ecology, Xiamen University, Xiamen, China; ^2^Southern Marine Science and Engineering Guangdong Laboratory (Zhuhai), Zhuhai, China; ^3^BGI-Shenzhen, Shenzhen, China

**Keywords:** particulate organic matter, prokaryotic community, metaproteomics, transporter, northwest Pacific Ocean

## Abstract

Solubilized particulate organic matter (POM) rather than dissolved organic matter (DOM) has been speculated to be the major carbon and energy sources for heterotrophic prokaryotes in the ocean. However, the direct evidence is still lack. Here we characterized microbial transport proteins of POM collected from both euphotic (75 m, deep chlorophyll maximum DCM, and 100 m) and upper-twilight (200 m and 500 m) zones in three contrasting environments in the northwest Pacific Ocean using a metaproteomic approach. The proportion of transport proteins was relatively high at the bottom of the euphotic zone (200 m), indicating that this layer was the most active area of microbe-driven POM remineralization in the water column. In the upper-twilight zone, the predicted substrates of the identified transporters indicated that amino acids, carbohydrates, taurine, inorganic nutrients, urea, biopolymers, and cobalamin were essential substrates for the microbial community. SAR11, Rhodobacterales, Alteromonadales, and Enterobacteriales were the key contributors with the highest expression of transporters. Interestingly, both the taxonomy and function of the microbial communities varied among water layers and sites with different environments; however, the distribution of transporter types and their relevant organic substrates were similar among samples, suggesting that microbial communities took up similar compounds and were functionally redundant in organic matter utilization throughout the water column. The similar vertical distribution of transport proteins from the euphotic zone to the upper twilight zone among the contrasting environments indicated that solubilized POM rather than DOM was the preferable carbon and energy sources for the microbial communities.

## Introduction

The global carbon cycle is strongly driven by the biological pump in the ocean, which involves a series of processes transferring carbon from surface waters to the ocean interior in the form of particulate organic matter (POM, >0.7 μm) (Jiao et al., 2010; Giering et al., 2014). During the sinking of POM throughout the water column, the solubilization rate of POM to dissolved organic matter (DOM, <0.7 μm) and the subsequent microbial uptake of cleavage products are considered to be the limiting steps of POM remineralization (Bergauer et al., 2018). It is reported that only approximately 10% of DOM directly satisfies the microbial carbon demand in the mesopelagic and bathypelagic realms (AríStegui et al., 2002), and solubilized POM and labile DOM are considered to be preferentially used (Baltar et al., 2009; Jiao et al., 2010). The solubilized POM in the oceanic water column represents DOM freshly solubilized from POM by microbes within the microenvironment of POM while the labile DOM, turned over by bacteria within days, is referred as the vast pool of DOM in the deep ocean (Bergauer et al., 2018). More and more evidences suggest that the labile DOM is not the major carbon and energy resources for microbial community in the deep sea (Baltar et al., 2009; Hansell et al., 2009; Bergauer et al., 2018) and less than 1% of the DOM in the ocean is labile and 94% is refractory (Hansell et al., 2009). In addition, a recent study suggests that the heterotrophic microbes (size between 0.2 and 0.8 μm) rely largely on solubilized POM rather than on autochthonous DOM as the carbon and energy sources in the mesopelagic and bathypelagic waters (Bergauer et al., 2018).

Transport proteins, located at the cell surface membranes as a channel or a carrying mechanism, are responsible for organic matter assimilation and nutrient uptake in microbes (Williams et al., 2012; Arséne-Ploetze et al., 2015). Transporter systems are regarded as the boundary that defines the interactions of cells with each other and with their surrounding environments (Tang et al., 2012). A recent study on microbial transporters shows that heterotrophic prokaryotes are geared toward the utilization of similar DOM compounds throughout the water column (Bergauer et al., 2018). However, metaproteomic and metagenomic studies suggest that the phylogenetic composition of the heterotrophic microbial community is depth stratified in the oceanic water column (Delong et al., 2006; Hawley, 2014; Tseng et al., 2015). Moreover, particle-attached microbes associated with POM and free-living microbes associated with DOM are taxonomically different and represent two distinct lifestyle strategies (Mestre et al., 2020). Direct evidence of utilizing solubilized POM by heterotrophic prokaryotes throughout the water column is still lacking due to less metabolic characterization of particle-attached microbial community. Furthermore, we know little about the utilization mechanism of the solubilized POM by heterotrophic prokaryotes in diverse marine environments.

In this study, to address these questions, we selected three contrasting environments in the northwest Pacific Ocean (Supplementary Figure 1 and Supplementary Table 1) and investigated the distribution patterns of microbial transport proteins in the POM collected from the euphotic zone and the upper twilight zone, the fastest microbial remineralization area of POM (Buesseler et al., 2007), using a metaproteomic approach. The environments of the three water columns presented significant differences due to the influences of the Kuroshio and Oyashio currents (Supplementary Table 1). Site K2, influenced by the Kuroshio Current, was characterized by a high temperature and low nutrient status, whereas site B1, influenced by the Oyashio Current, was featured by a low temperature and high nutrient status. Site B9 was located at the confluence of the Kuroshio Current and Oyashio Current, and therefore the temperature and nutrient status fell between those of the two other sites. We characterized composition, origin, and vertical distribution of transport proteins of particle-attached microbial communities (0.7–200 μm) throughout the water column. Our results indicated that the heterotrophic prokaryotic community was inclined to utilize similar substrates throughout the water column even in the contrasting environments. Moreover, the vertical distribution of transport proteins from the euphotic zone to the upper-twilight zone indicated solubilized POM rather than DOM as a preferable carbon and energy source for microbes.

## Materials and Methods

### Cruise Information and Sample Collection

A multidisciplinary cruise was conducted in the northwest Pacific Ocean from March 30 to May 10, 2015, onboard the RV *DongFangHong* 2. POM samples (size between 0.7 and 200 μm) for metaproteomic analysis were collected from three sites: K2 (134°00′E, 25°00′N), B1 (147°00′E, 38°00′N) and B9 (147°00′E, 30°00′N) (Supplementary Figure 1). POM-associated microbes from four depths of three sites, K2: 75, 125 m (deep chlorophyll maximum, DCM), 200 and 500 m, B1: DCM (50 m), 100, 200, and 500 m, and, B9: DCM (25 m), 100, 200, and 500 m, were *in situ* captured using a large volume water transfer system (McLane Research Laboratories, Inc., United States) (Supplementary Table 1). A range of 800–1,000 L seawater was filtered as one of the biological duplicates for each sample via two pumps simultaneously deployed at the same depth. During filtration, seawater was pre-filtered through a 200 μm mesh filter and then retained on a GF/F membrane (142 mm in diameter, Millipore Corporation, MA, United States). The GF/F membranes containing POM were stored at −80°C until analysis. The temperature, salinity, depth, chlorophyll fluorescence and dissolved oxygen values at each site were retrieved from a conductivity-temperature-depth rosette system (CTD, Sea Bird Electronics, United States). Seawater samples for inorganic nutrient (NO_3_^–^, NO_2_^–^, SiO_3_^2–^, and PO_4_^3–^) analysis were filtered through a 0.22 μm pore size polycarbonate membrane and immediately analyzed onboard with an AA3 automatic continuous flow analyzer (Seal AA3, Norderstedt, Germany). The detection limits for the NO_3_^–^, NO_2_^–^, SiO_3_^2–^, and PO_4_^3–^ concentrations were 0.1, 0.1, 0.2, and 0.05 μM, respectively. NH_4_^+^ was measured on board using a fluorometric method with detection limit of 1.2 nmol L^–1^ and precision of ±3.5% (Zhu et al., 2013). Urea was measured using a 1 m-long liquid waveguide capillary cell based on the colorimetric reaction with diacetyl monoxime with detection limit of 1 nmol L^–1^ (Chen et al., 2015). POC and particulate organic nitrogen were analyzed using a Thermo Finnigan Flash EA1112 elemental analyzer (Thermo Electron Corp., Waltham, MA, United States) with acetanilide as the calibration standard.

### Protein Extraction and Identification

The filter membranes were thawed and cut into chips (2 × 2 mm) using a sterilized razorblade. The membrane chips (per gram) were suspended in 20 ml of Trizol reagent (Ambion-Life Technologies, United States) and disrupted three times in a FastPrep-24 homogenizer (MP Biomedicals, Santa Ana, CA, United States), each for 20 s. Then, the mixtures were centrifuged at 20,000 × *g* for 30 min after incubation at 25°C for 1 h. The supernatants were subsequently used for protein extraction following the Trizol Reagent manufacturer’s protocol. A rehydration buffer containing 7 M urea, 2 M thiourea and 4% (w/v) CHAPS was added to dissolve the protein pellets. The protein samples were then centrifuged at 14,000 × *g* to remove cell debris, and the protein concentrations in the supernatants were determined using a 2D Quant Kit (GE Healthcare, United States). Buffer exchange was performed with 100 mM of NH_4_HCO_3_ using a 10 kDa Amicon filter (Millipore, MA, United States). The purified protein samples (100 g) were subjected to overnight Trypsin digestion and were separated on C18 (Sigma-Aldrich, United States) and strong cation exchange solid-phase extraction columns. Each digested peptide sample was loaded on an UltiMate 3000 UHPLC system (Thermo Fisher Scientific, Waltham, MA, United States) equipped with a 300 μm i.d. × 0.5 cm C18 TRAP column (μ-Precolumn, Thermo Fisher Scientific, Waltham, MA, United States) and a 75 μm i.d. × 25 cm analytical column packed in-house with 3 μm C18 particles, then was injected into mass spectrometry (MS) twice for technical repeats using a Q Exactive HF hybrid quadrupole-Orbitrap mass spectrometer (Thermo Fisher Scientific, Waltham, MA, United States). The MS parameters were set as follows: spray voltage 2 kV, capillary temperature 320°C, positive mode, scan range of 350–1,500 *m*/*z*, loop count 30, NCE 26, MS resolution 120,000, MS/MS HCD scan resolution 30,000 and dynamic exclusion duration 30 s. Each fraction was injected twice for confident identification.

The MS raw data of each sample were merged and formatted to MGF files using Proteome Discoverer (ver. 1.3.0.339; Thermo Fisher Scientific, San Jose, CA). A global Ocean Microbial Reference Gene Catalog (OM-RGC) dataset was used for MS/MS database search, considering its maximum coverage of microbial genetic diversity in the epipelagic and mesopelagic waters across the globe, including north and south Pacific Ocean (Sunagawa et al., 2015). For better interpretation of MS/MS spectra, a three-step iterative search was applied for protein identification and quantification using the MetaPro-IQ approach (Zhang et al., 2016). Briefly, the first- and second-step database searches against the OM-RGC database were performed to generate a reduced database that contained all possible proteins derived from peptide-spectrum matches (PSMs) for all samples using X!Tandem software (2017.2.1 version). The reduced database containing the resulting protein lists was then imported into MaxQuant software (1.6.1.0 version) for protein quantification (Cox and Mann, 2008; Maier and Wong, 2015). In the first- and second-step database searches, the tandem search was performed with up to one miss-cleavage (trypsin/P), carbamidomethylation of cysteine as a fixed modification, and oxidation of methionine as a potential modification. A fragment ion tolerance of 20 ppm and a parent ion tolerance of 0.05 Da were used. All matched protein sequences for the first-step search were extracted as the sample-specific database (S_all-macth.fasta). The X!Tandem outputs of the target-decoy database search (step 2) were summarized with in-house software to generate an identified protein list at a PSM false discovery rate (FDR) cutoff of 0.01. The resulting protein list for all samples was then combined, and duplicates were removed to generate a “combined non-redundant database” for protein quantification using MaxQuant software (1.6.1.0 version). Similar peptide identification parameters with X! Tandem database searches were used for MaxQuant search. To reduce the probability of false peptide identification, the FDR was set to less than 1% at both PSM and protein levels. For PSM and protein quantification, the label-free quantification (LFQ) algorithm was used. Both razor and unique peptides were used for protein quantification, and the minimum ratio count was set as 1. An alignment retention time window of 20 min and a match time window of 5 min were applied to match the same accurate masses between different runs. High-confidence proteins matching at least two PSMs and one unique peptide were selected for further analysis.

Proteins identified by the same set or a subset of peptides were grouped together as one protein group. Leading proteins (defined as the top ranking proteins in a group; ranking was based on the number of peptide sequences, the number of PSMs and the sequence coverage) were selected for further taxonomic and functional analysis (Zhang et al., 2016).

### Taxonomic and Transport Substrate Annotation of Proteins

The leading proteins were used for taxonomic and functional analysis. The leading proteins were searched against the NCBI non-redundant protein database (NCBInr, 2017_9) using BLASTP, and the top 10 hits were recorded. The BLASTP search results were loaded into MEGAN (Huson et al., 2007), and the taxonomic assignment was performed using the lowest common ancestor algorithm (bit score > 80) (Huson et al., 2011). All the quantified protein sequences were aligned against (i) the Clusters of Orthologous Groups (COGs) database (90.97% of total proteins)^1^ (Galperin et al., 2014), and (ii) the Kyoto Encyclopedia of Genes and Genomes (KEGG) database (96.63% of total proteins) (Kanehisa et al., 2016) with BLASTP, using default parameters (*e*-value cut-off of 0.00001). Transporters were manually examined and selected, and their predicted substrates were extracted according to the COG and KEGG annotations.

### Data Analysis

Statistical analyses and graphs were performed using the R programming language^2^. The samples were categorized according to the station and depth. Proteins of each sample were selected if they were identified in at least two duplicates, and the relative abundance of each protein was averaged among biological replicates. A permutational multivariate analysis of variance (PERMANOVA) (Adonis test, R package VEGAN) was performed to discern statistically significant difference in community structure explained by different factors: size-fraction, station, and depth. For proteome-based community structure analysis, the relative abundance of each microbial group was calculated by summing its total protein abundances and then divided by the sum of all protein abundances in a sample (community-level analysis). For function comparison, the relative abundance of each protein and metabolic KEGG pathway was calculated by summing the related protein abundances and then divided by the sum of all protein abundances in a species (species-level analysis) (Zhang et al., 2019). An analysis of variance (ANOVA) was conducted to test whether the distribution (i.e., the relative transport protein abundances, percentage contribution to the community) of each taxonomic group along the substrate types was conserved at the various stations and depths.

## Results and Discussion

### Overview of Microbial Transport Proteins

12 POM samples from both euphotic and upper-twilight zones were collected in three contrasting environments (sites K2, B1, and B9, Supplementary Table 1 and Supplementary Figure 2) in the northwest Pacific Ocean (Supplementary Figure 1). A total of 48 samples each containing two biological replicates and two technological replicates were subjected to proteomic analysis. A three-step search strategy was applied to improve the protein identification and biological and technological repeated samples were clustered together (Supplementary Figure 3), indicating high quality of the metaproteome data. A total of 24,967 proteins with quantitative information were identified, of which proteomic coverage were comparable or better than previously reported in other oceanic regions (Morris et al., 2010; Williams et al., 2012; Georges et al., 2014). Notably, the proteins identified among the three sites differed significantly (Supplementary Tables 2, 3), and only 7,330 proteins were shared among the three sites (Supplementary Figure 4). The taxonomic and functional structures of the microbial community also differed in each layer (Supplementary Figures 5, 6). Based on previous studies of both particle-attached and free-living microbes in different regions (Zhang et al., 2007; Li et al., 2015; Mestr et al., 2017; Mestre et al., 2017; Gómez-Consarnau et al., 2019; Sebastián et al., 2019; Mestre et al., 2020), as well as a study on particulate-derived experimental isolates (Enke et al., 2018), the particle-attached microbes accounted for the greater proportion, up to 73.69% in our data. In addition, pilin proteins (based on KEGG functional annotation), which constitute the defining characteristic of particle-attached microbes (Maier and Wong, 2015), were detected in the dominant microbial groups, further demonstrating that these bacteria were particle attached.

Among all proteins, 5,696 transport proteins (22.81% of the abundance) were annotated based on the COG and the KEGG Orthology (KO) databases. The taxonomic diversity derived from transport proteins, referring to the substrate-active community, mainly comprised bacteria (5,275 proteins, 92.92% of the abundance), Eukaryota (209 proteins, 4.60%) and Archaea (106 proteins, 1.05%) (Supplementary Table 3). The percentage of transport proteins relative to all proteins identified was low in the DCM layer of the three sites, while the proportion of transport proteins from eukaryotes was the highest (Supplementary Table 3). Notably, the abundances of transport proteins were much higher around the bottom of euphotic zone (depths of 200 m at site K2, 100 m at sites B1 and B9). These protein indicators of substrate uptake implied highly active remineralization driven by POM-associated microbes in this region. Although the sharp decline of POC (Supplementary Table 1) might be mainly caused by the decrease of POC generation, the POC flux of the water column in the Pacific Ocean confirms that these two layers have a higher respiration rate than other water layers (Buesseler et al., 2009; Giering et al., 2014).

**FIGURE 1:**
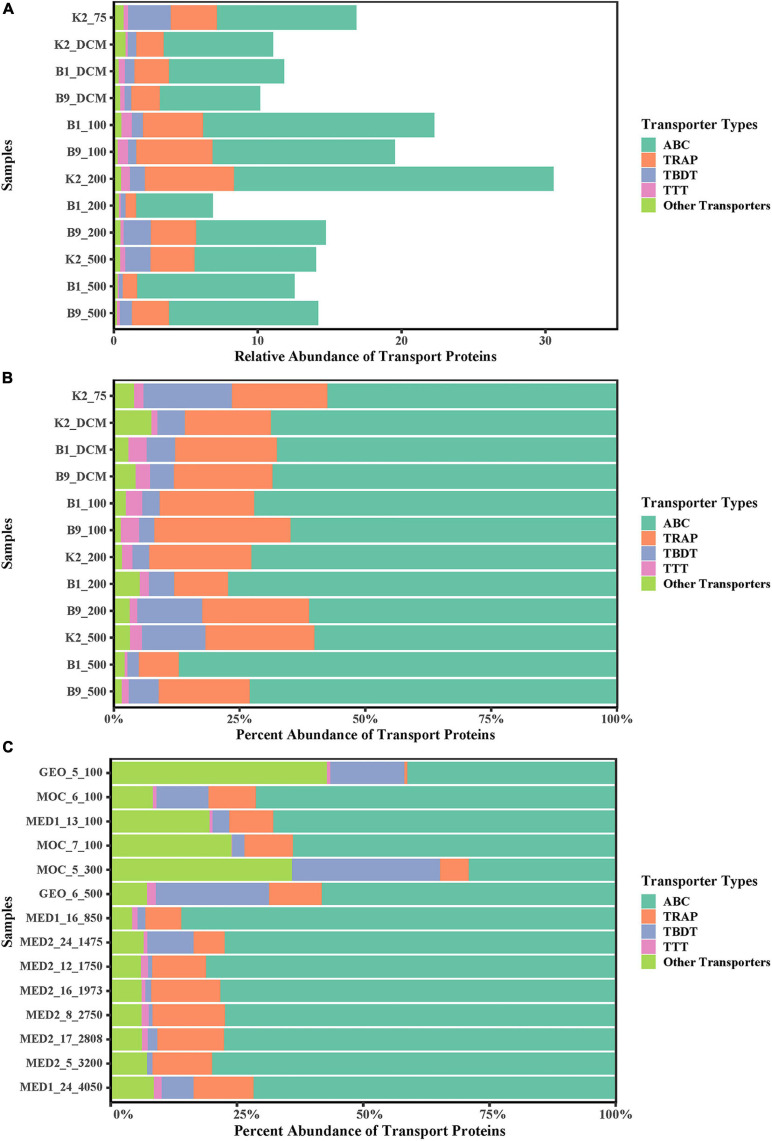
Vertical distribution and relative abundance ratio of ABC, TRAP-T, TTT, and TBDT in all proteins **(A)** And all transport proteins **(B)** Of POM solubilization in northwest Pacific Ocean. **(C)** Vertical distribution and relative abundance ratio of ABC, TRAP-T, TTT, and TBDT in all transporters proteins of DOM in Atlantic Ocean (Bergauer et al., 2018). ABC, ATP-binding cassette; TRAP-T, tripartite ATP-independent; TTT, tripartite tricarboxylate; TBDT, TonB-dependent. Other Transporters, other type of transport proteins. Relative abundances of each transporter type were calculated by summing abundances of all protein assigned to the same type. The percent abundance of transport proteins was calculated by summing the related transport protein abundances and then dividing by the sum of all transport protein abundances.

In this study, we focused on the transport proteins from bacteria and archaea due to their important roles in the utilization of solubilized POM. Based on the label-free quantitative proteomics results, the profiles of transport proteins showed functionally similar transport processes in all samples regarding their relative abundances. Similar to previous metaproteomic studies (Morris et al., 2010; Sowell et al., 2011; Williams et al., 2012; Georges et al., 2014; Colatriano et al., 2015; Bergauer et al., 2018), ATP-binding cassette (ABC) transporters were the most prevalent transporter system in our metaproteome data, and the tripartite ATP-independent periplasmic (TRAP), TonB-dependent receptor (TBDT), and tripartite tricarboxylate (TTT) transporters also presented relatively high abundances in the three water columns (Figure 1). The substrate-binding proteins (SBPs), which are the primary determinants of selectivity in import systems (Berntsson et al., 2010; Lewinson and Livnat-Levanon, 2017), were the most frequently encountered components of the ABC complex (3,099 proteins, 84.76% of ABC transporter proteins). For bacteria and Archaea, all SBP-dependent ABC systems are importers and ubiquitous. SBPs are constituents of primary and secondary active transporters and capture substrates with high affinity from the cell surroundings (Berntsson et al., 2010; Lewinson and Livnat-Levanon, 2017), and play an important role in nutrient uptake (Saier, 1998). The structure of the transporter patterns of POM was similar in different layers and environments, which differed from the previous metaproteomic study on DOM in which the structure of transporter patterns differs between the epipelagic and mesopelagic zones, and the abundance of TBDTs in the DOM is higher in the mesopelagic zone than in the epipelagic zone (Bergauer et al., 2018). In their data, the relative abundance of TBDTs in the Atlantic Ocean had a higher variability than that in the three water columns (coefficient of variation: 1.19 vs. 0.63). TBDTs play an important role in the uptake of high-molecular-weight organic matter (Mccarren et al., 2010), which makes up an important part of the labile DOM (Benner et al., 1992). The increase of TBDT abundance in the DOM from the epipelagic zone to the mesopelagic zone in the Atlantic Ocean might be interpreted as an adaptation of the microbial community to the change in the labile DOM (Bergauer et al., 2018).

### Vertical Substrate Uptake Patterns Based on Transport Proteins

**FIGURE 2:**
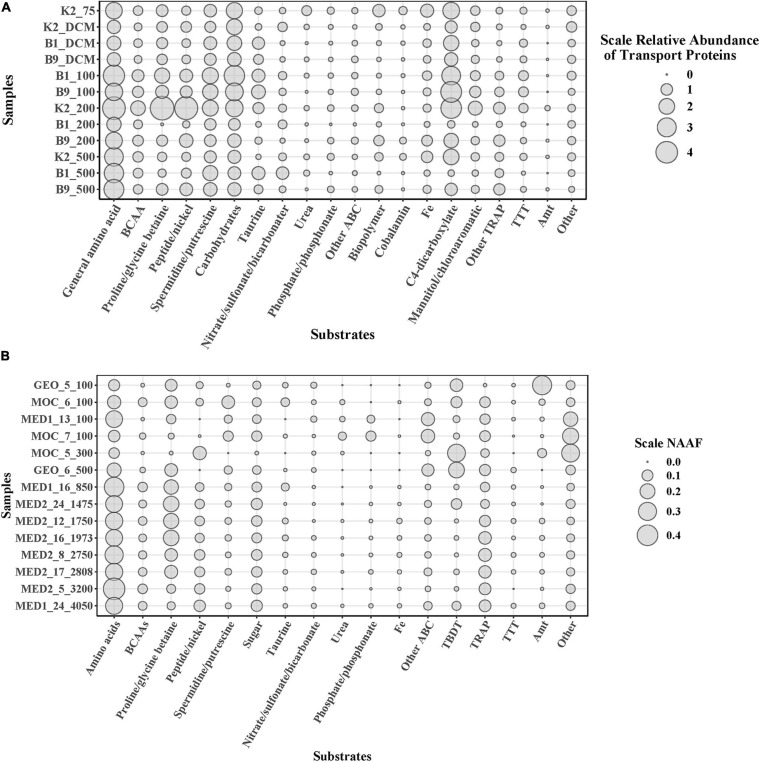
Vertical expression profiles of selected transporters of POM solubilization in northwest Pacific Ocean **(A)** And transporters of DOM in Atlantic Ocean **(B)** Analyzed based on NAAF values (Bergauer et al., 2018). Transporter proteins were grouped by the predicted substrate specificity of the substrate-binding proteins. BCAAs, branched-chain amino acids; NAAF, normalized area abundance factors.

To elucidate microbial metabolic features throughout the water column, we further predicted substrates uptaken by the microbial community in the POM. Diverse substrates of transport proteins were predicted, such as amino acids, carbohydrates, taurine, inorganic nutrients, urea, biopolymers and cobalamin (Figure 2), similar to previous studies that focus on the transporters of DOM (Morris et al., 2010; Bergauer et al., 2018). Although the components of DOM and solubilized POM are different, heterotrophic prokaryotes utilize similar substrates from them. Furthermore, combined with previous studies (Morris et al., 2010; Bergauer et al., 2018), different microbial community structures presented similar substrate uptake patterns in different depths and oceans. Taken together, these results indicate that the essential components of the organic matter pool available for the microbial community might be similar in the global ocean.

**FIGURE 3:**
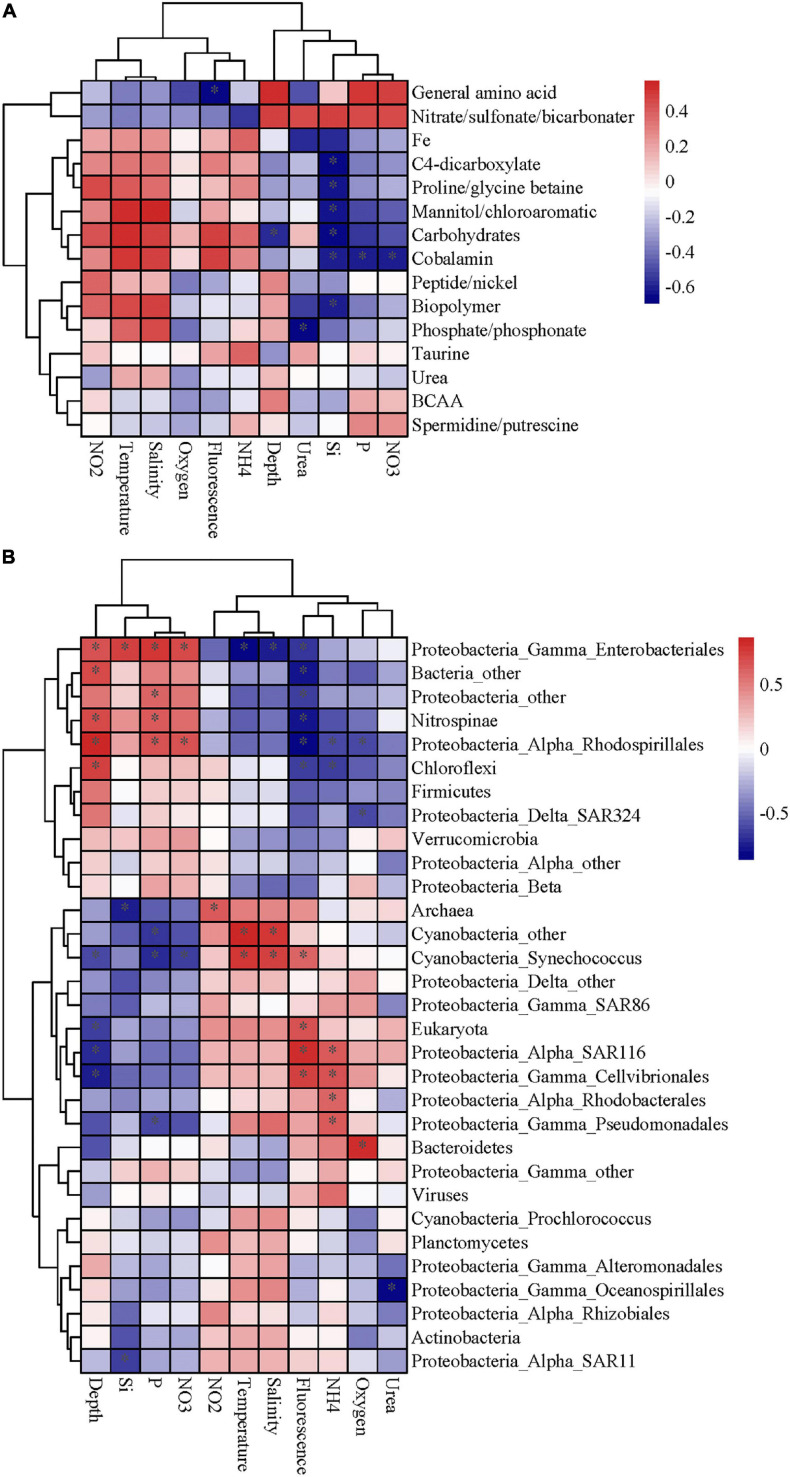
The Spearman correlation analysis was performed to determine the correlation between environmental factor and substrate or microbial community. **(A)** The correlations between substrates and environmental factors. **(B)** The correlations between substrate-active community and environmental factor. The fluorescence represented chlorophyll fluorescence. The color of the boxes show the correlation coefficient. The “*” represents statistical significance with *p* ≤ 0.05.

The expression patterns of prevalent transporter systems targeting various substrates exhibited an insignificant difference regarding the substrate affinity of transporter systems (Figure 2). Furthermore, the correlation between substrates and environmental factors also indicated that the microbial utilization of substrates was insignificantly affected by depth and most of the environmental factors (*p* > 0.05) (Figure 3A). Interestingly, the low nutrient status at site K2 had little impact on the microbial utilization of inorganic nitrogen or phosphate. In the three contrasting water columns, ABC transporters exhibited the highest relative abundance in each layer owning to their involvement in the uptake of amino acids, carbohydrates, and taurine. This is in agreement with a previous study that amino acid-like compounds contribute most (40–50%) to POC, with roughly 30% carbohydrate and 20% lipid in the remaining mass (Hedges et al., 2001). Various proteinogenic amino acids were predicted, such as glutamate, arginine and derivatives, histidine, proline, and glycine or more general branched-chain amino acids (leucine, isoleucine, and valine), and polar- and L-amino acids (Supplementary Table 3). The abundance of carbohydrate transporters was comparable with that of peptide transporters (Figure 2). The relative abundance of dipeptide transporters was twice as high as that of oligopeptides, independent of the sampling depth. The ABC transporters with predicted substrates of nitrate/sulfonate/bicarbonate, urea, phosphate/phosphonate, organic compounds containing carbon, nitrogen, or sulfur moieties presented considerably low abundance. Notably, the taurine transporter presented a relatively high abundance in our study, but its abundance is relatively low in the previous study (Bergauer et al., 2018). Taurine constitutes an important food source for heterotrophic microbes due to its carbon, nitrogen, sulfur moieties, and energy source, and it is found at nanomolar concentrations in marine environments (Clifford et al., 2017, 2019). Interestingly, taurine is reported as DOM and is utilized by free-living bacteria, such as SAR11 and SAR116 (Sowell et al., 2011; Williams et al., 2012; Hawley, 2014). However, in our study, most taurine transporters (>52%) were detected in Gammaproteobacteria which are considered as particle-attached microbes. Taurine has been reported in a broad range of phytoplankton species (Tevatia et al., 2015), and phytoplankton is the important component of POM. These results suggest that taurine might be mainly utilized by particle-attached microbes. Moreover, diverse microbial groups had relatively high abundances of TRAP transporters, which are ATP independent and thus less energy consuming than ABC transporters. Predicted substrates of TRAP identified in this study included mannitol/chloroaromatic compounds, aromatic acids, and C4-dicarboxylates such as malate, fumarate, and succinate. Among them, the uptake of C4-dicarboxylates was more active. TBDT activities were once thought to be restricted to iron complexes (siderophores), and vitamin B12 (cobalamin), but a recent study indicates that nickel, cobalt, copper, maltodextrins, sucrose, thiamin, and chito-oligosaccharides are also substrates for TBDTs (Morris et al., 2010). In our study, TonB-dependent transporters were mainly assigned to the utilization of Fe, which is a limiting nutrient for microbes and plays an important role in carbon cycle (Blain et al., 2007). Notably, the ammonium channel protein (amt) transporter presented a rather low abundance (0.14%) in our study, but it was relatively abundant in the previous study on DOM utilization (3.74%) (Bergauer et al., 2018). Considering the fact that particle-attached microbes are often found in POM and free-living counterparts in the DOM, the low abundance of amt transporter herein suggests that the microbial groups assigned to amt transporters are mainly free-living but rather particle-attached. Around 73% of amt transporters were from Thaumarchaea and the rest from Actinobacteria, which confirms our hypothesis.

### The Substrate-Active Microbial Community

**FIGURE 4:**
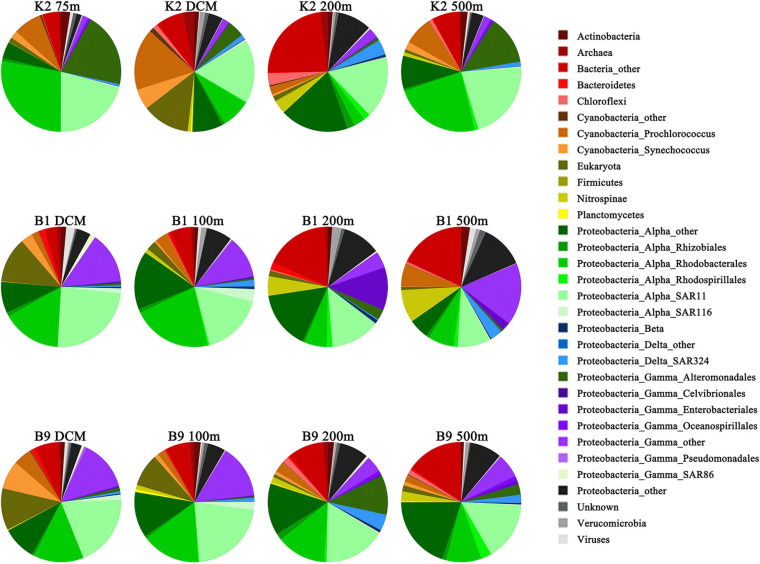
Taxonomic composition of prokaryote communities identified in the transport proteins. Prokaryotic taxa with >1% relative abundance on average are displayed and named in the format, phylum_class_order.

The substrate-active microbial community was comprised four main phyla: Proteobacteria (Alphaproteobacteria, 45.85% of the abundance; Gammaproteobacteria, 4.87%; Deltaproteobacteria, 1.82%), Cyanobacteria (7.10%), Nitrospinae (2.10%), and Actinobacteria (1.50%) (Figure 4). At the order level, SAR11 (17.67%), Rhodobacterales (13.74%), Alteromonadales (4.87%), and Enterobacteriales (1.30%) were the key contributors to the transporters. The taxonomic distribution of transporters was similar at the kingdom level among different layers and sites but showed large differences at the phylum and order levels (Figure 4). Among the three sites, the dominant microbial groups in the DCM layer at site K2 were *Prochlorococcus*, SAR11 and Rhodobacterales, while SAR11, Rhodobacterales, and Enterobacteriales were more abundant at sites B1 and B9. In a previous study, *Prochlorococcus* transport proteins are detected at low-nutrient extremes in the Sargasso Sea (Sowell et al., 2009), which is consistent with our results. SAR11 and Rhodobacterales are aerobic heterotrophs who can scavenge dissolved organic carbon and nutrients under the oligotrophic conditions of the open ocean and have diverse lineages at high abundance in water columns based on gene surveys (Vergin et al., 2013; Tsementzi et al., 2016; Enke et al., 2018). Alteromonadales were predominant only in the 200 m layer at site B9, which was characterized by a relatively high concentration of nitrite, ammonium and urea (Supplementary Table 1). A depth-dependent stratification of substrate-responsive phyla based on the expression of transporter proteins was shown by Cyanobacteria, Actinobacteria, Bacteroidetes, Verrucomicrobia, Firmicutes, and the candidate phylum Marinimicrobia*;* they exhibited higher relative abundances in the euphotic zone than in the upper twilight zone (Figure 4). Conversely, transporters from Deferribacterales, Planctomycetes, Chloroflexi and members of Gammaproteobacteria and Deltaproteobacteria were relatively more abundant in the upper twilight zone than in the euphotic zone. The correlations between the substrate-active microbial community and environmental factors suggested that the substrate-active microbial communities were influenced by many environmental factors, including nutrients, temperature and salinity (*p* < 0.05) (Figure 3B). Enterobacteriales, Nitrospinae, and Rhodospirillales were more likely impacted by depth and concentrations of silicate, phosphate and nitrate (*p* < 0.05), and the SAR116, Cellivibrionales, Rhodobacterales, and Psudomonadales were influenced by ammonia (*p* < 0.05), whereas *Cyanobacteria*, especially *Synechococcus* were affected by temperature and salinity.

### Substrate Uptake Patterns at the Community Level

Various bacterial and archaeal phyla presented different abundances in each depth layer according to their involvement in the uptake of different substrates. With respect to the uptake of amino acids, Alpha- and Gamma-Proteobacteria exhibited the highest abundance in all layers, contributing ∼45 and 67% to the euphotic and upper twilight zones, respectively. Among them, the SAR11 clade had a relatively high abundance in all layers in the uptake of compatible solutes and inorganic matter to support cell growth. Moreover, the SAR11 clade members appeared to be the primary utilizer of taurine in the upper twilight zone, in agreement with previous studies (Williams et al., 2012; Lewinson and Livnat-Levanon, 2017). The profiles of the active community involved in carbohydrate utilization indicated a vertical stratification in the water column, with higher relative abundances of *Prochlorococcus*, Rhodobacterales, and SAR11 in the euphotic zone, whereas other Gammaproteobacteria dominated in the upper twilight zone. The SAR324 clade members contained a high abundant transporter specific to proline/glycine betaine as opposed to glycerol-3-phosphate, which is also relatively abundant in the previous study on DOM utilization (Bergauer et al., 2018). The taxonomic classification and relative abundance of TBDTs throughout the water column revealed a clear stratification of Flavobacteria, Alteromonadales, SAR86 cluster bacteria and Marinimicrobia.

**FIGURE 5:**
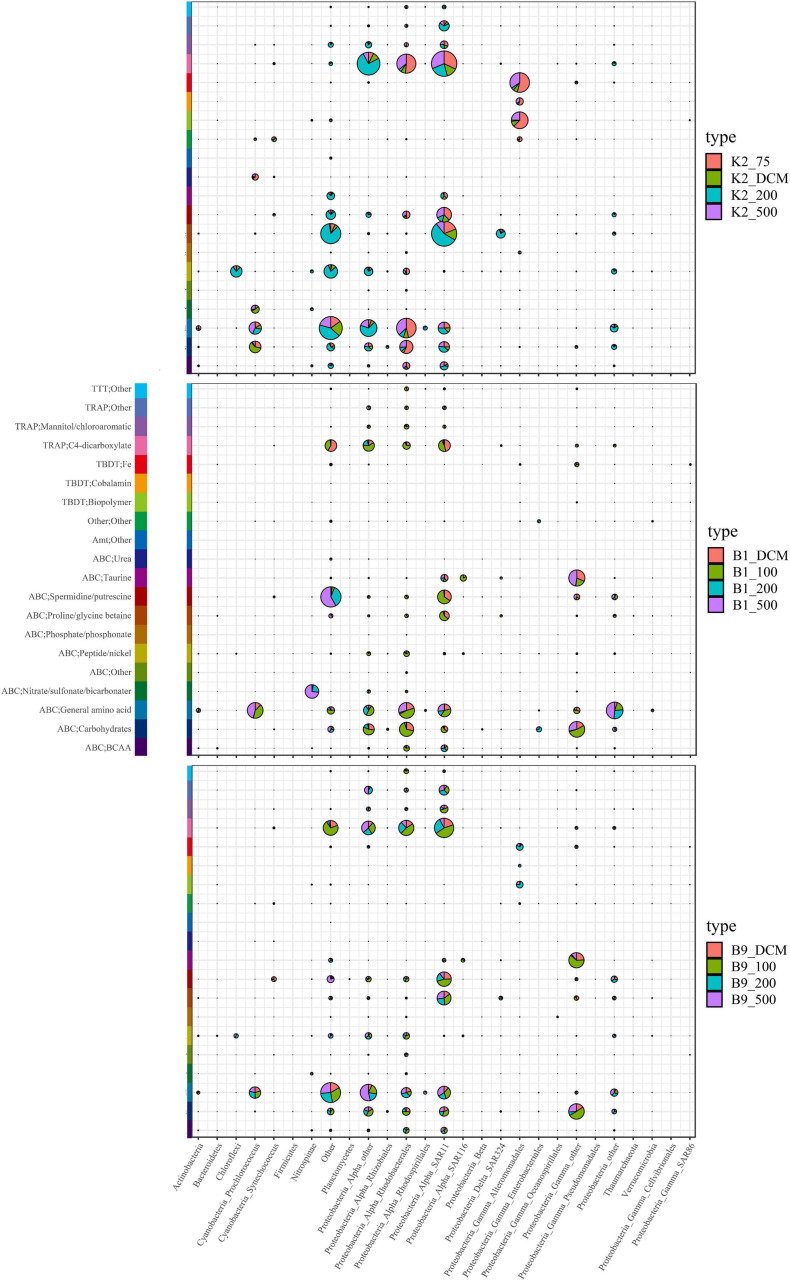
Vertical expression profiles of transporter proteins of abundant taxa. Expression values were calculated in a semiquantitative manner and average abundances were plotted for selected members of the substrate active community residing in the water layers.

Interestingly, our study found that different taxonomic groups were assigned to the same substrates in the same layers at the three sites (Figure 5), indicating that the same substrates were consumed by different microbial groups. For example, carbohydrates and amino acids were transported and utilized by *Prochlorococcus* in the DCM layer at site K2; however, the main consumer was Alphaproteobacteria at sites B1 and B9. Furthermore, different taxonomic distributions assigned to the same substrates often occurred in the same class but different genera. A similar result was also observed in the transport of iron: Alteromonadales at site K2 and Gammaproteobacteria at sites B1 and B9 were responsible for iron transport. In addition, different microbial groups used similar substrates at different depths. In particular, the origin of the species responsible for the substrate utilization presented a significant distinction in either the euphotic zone or the twilight zone. For example, the transport and utilization of carbohydrates was carried out by Alphaproteobacteria in the euphotic zone at site B1; however, this process was carried out by Enterobacteria in the upper twilight zone.

## Conclusion

In summary, our study revealed similar vertical distribution patterns of prevalent transporter systems in the POM throughout the water column in three contrasting environments. ABC, TRAP, TBDT, and TTT were four major transporters responsible for transporting various substrates. SAR11, Rhodobacterales, Alteromonadales, and Enterobacteriales were the key contributors to transporters. The heterotrophic prokaryotic communities were inclined to utilize similar organic substrates throughout the water column via the same transport systems. Furthermore, the similar vertical distribution of transporters from the euphotic zone to the upper twilight zone also supported the speculation that allochthonous DOM solubilized from POM rather than autochthonous DOM is the main carbon and energy resources in the dark ocean. Future efforts should be devoted to microbial transport proteins of POM from diverse oceanic environments to verify our findings. In addition, the isolation of distinct microbial groups attached to or associated with POM, and subsequently studying their utilization of POM under laboratory conditions will deepen our understanding of the microbial remineralization mechanism of POM in the ocean.

## Data Availability Statement

The original contributions presented in the study are publicly available. This data can be found here: ProteomeXchange repository, accession number: PXD014630 (http://proteomecentral.proteomexchange.org/cgi/GetDataset?ID=PXD014630).

## Author Contributions

D-ZW and L-FK conceived the study. L-FK and LL collected and measured the samples. L-FK, Y-BH, Z-XX, K-QY, and H-KX analyzed the data. L-FK, Y-BH, Z-XX, S-QL, and D-ZW contributed to the discussion of the results. L-FK, Y-BH, Z-XX, and D-ZW wrote the manuscript. All authors contributed to the article and approved the submitted version.

## Conflict of Interest

K-QY, Y-BH, H-KX and S-QL were employed by company BGI-Shenzhen. The remaining authors declare that the research was conducted in the absence of any commercial or financial relationships that could be construed as a potential conflict of interest.

## Footnotes

1 ftp://ftp.ncbi.nih.gov/pub/COG/COG2014/data

2 www.r-project.org
