# Supplementary material for: Metaproteomics Reveals Similar Vertical Distribution of Microbial Transport Proteins in Particulate Organic Matter Throughout the Water Column in the Northwest Pacific Ocean
Source: Front Microbiol. 2021 Mar 25;12:629802. doi: 10.3389/fmicb.2021.629802 (PMC8034268; doi:10.3389/fmicb.2021.629802)
Supplement: Supplementary file 1 [file Data_Sheet_1.docx]

Supplementary Material

# Supplementary Table

**Supplementary Table 1.** Environmental data from Northwest Pacific Ocean sample sites.

|  | **K2** | | | **B1** | | | **B9** | | |
| --- | --- | --- | --- | --- | --- | --- | --- | --- | --- |
| Date | 5 April 2015 | | | 26 April 2015 | | | 10 April 2015 | | |
| Location | 134º00´ E, 25º 00´N | | | 147º00´ E, 38º 00´N | | | 147º00´ E, 30º 00´N | | |
| DCM depth | 125m | | | 50m | | | 25m | | |
|  | ***Physical properties*** | | | | | | | | |
|  | Temp. (°C) | Sal. | Fluo. | Temp. (°C) | Sal. | Fluo. | Temp. (°C) | Sal. | Fluo. |
| 75m | 20.63±0.05 | 34.92±0.15 | 0.1814±0.01 | - | - | - | - | - | - |
| DCM | 20.27±0.12 | 34.90±0.23 | 0.3131±0.06 | 12.98±0.15 | 34.29±0.42 | 0.5882±0.13 | 18.62±0.15 | 34.85±0.17 | 0.1449±0.01 |
| 100m | - | - | - | 10.66±0.06 | 34.23±0.23 | 0.0152±0.01 | 17.95±0.26 | 34.81±0.30 | 0.3519±0.02 |
| 200m | 18.37±0.07 | 34.78±0.09 | 0.0611±0.01 | 6.06±0.05 | 33.63±0.14 | 0.0547±0.01 | 17.33±0.08 | 34.77±0.26 | 0.0532±0.01 |
| 500m | 10.84±0.07 | 34.30±0.14 | 0.0545±0.01 | 8.36±0.05 | 34.04±0.15 | 0.0573±0.01 | 12.29±0.07 | 34.38±0.18 | 0.0835±0.01 |
|  | ***Physical properties*** | | | | | | | | |
|  | O_2_ (μM) | PON (mmol/L) | POC (mmol/L) | O_2_ (μM) | PON (mmol/L) | POC (mmol/L) | O_2_ (μM) | PON (mmol/L) | POC (mmol/L) |
| 75m | 682±1.53 | 0.37±0.05 | 2.45±0.17 | - | - | - | - | - | - |
| DCM | 678±2.89 | 0.19±0.04 | 1.67±0.16 | 825±3.59 | 1.08±0.31 | 6.05±0.85 | 692±1.83 | 0.41±0.03 | 2.66±0.61 |
| 100m | - | - |  | 733±2.66 | 0.21±0.01 | 1.12±0.26 | 668±2.11 | 0.15±0.01 | 0.74±0.01 |
| 200m | 599±0.85 | 0.08±0.04 | 0.43±0.05 | 858±5.27 | 0.23±0.01 | 0.41±0.08 | 682±1.64 | 0.11±0.01 | 0.40±0.01 |
| 500m | 508±0.71 | 0.06±0.06 | 1.22±0.08 | 269±1.23 | 0.14±0.01 | 0.76±0.12 | 54±0.73 | 0.10±0.01 | 0.75±0.02 |
|  | ***Nutrients (μM)*** | | | | | | | | |
|  | PO_4_^3-^ | SiO_3_^2-^ | NO_3_^−^ | PO_4_^3-^ | SiO_3_^2-^ | NO_3_^−^ | PO_4_^3-^ | SiO_3_^2-^ | NO_3_^−^ |
| 75m | 0.11±0.01 | 0.5±0.01 | 0.15±0.01 | - | - | - | - | - | - |
| DCM | 0.16±0.01 | 12.96±1.17 | 2.26±0.05 | 0.41±0.01 | 7.92±0.51 | 4.82±0.17 | 0.13±0.01 | 20.9±0.71 | 0.12±0.01 |
| 100m | - | - | - | 0.78±0.02 | 15.56±1.14 | 9.5±0.40 | 0.25±0.01 | 10.03±2.43 | 2.29±0.37 |
| 200m | 0.27±0.01 | 2.11±0.41 | 3.66±0.11 | 2.66±0.06 | 83.57±1.28 | 36.65±3.73 | 0.19±0.01 | 2.2±0.42 | 2.22±0.25 |
| 500m | 1.34±0.34 | 26.86±0.33 | 19.21±0.73 | 2.67±0.04 | 84.53±1.06 | 36.62±2.05 | 2.66±0.36 | 84.25±3.03 | 36.73±2.29 |
|  | ***Nutrients (μM)*** | | | | | | | | |
|  | NO_2_^−^ | NH_4_^+^ | Urea | NO_2_^−^ | NH_4_^+^ | Urea | NO_2_^−^ | NH_4_^+^ | Urea |
| 75m | 0.031±0.01 | 0.0863±0.01 | 0.0855±0.01 | - | - | - | - | - | - |
| DCM | 0.204±0.01 | 0.0421±0.01 | 0.1242±0.01 | 0.213±0.01 | 0.0712±0.01 | 0.1041±0.01 | 0.017±0.01 | 0.0614±0.01 | 0.1023±0.01 |
| 100m | - | - | - | 0.027±0.01 | 0.0761±0.01 | 0.1086±0.01 | 0.124±0.01 | 0.0479±0.01 | 0.1175±0.01 |
| 200m | 0.043±0.01 | 0.0349±0.01 | 0.0525±0.01 | 0.026±0.01 | 0.0434±0.01 | 0.1094±0.01 | 0.161±0.01 | 0.0613±0.01 | 0.0652±0.01 |
| 500m | 0.02±0.01 | 0.0375±0.01 | 0.0528±0.01 | 0.02±0.01 | 0.0462±0.01 | 0.1091±0.01 | 0.03±0.01 | 0.0472±0.01 | 0.0515±0.01 |

Triplicate samples were collected for each environmental factor. Abbreviation: Temp. is temperature, Sal. is salinity, and Flou. is Flourescence.

**Supplementary Table 2.** The results of protein identification.

| Group | Sample numbers | Total spectra | Identified spectra | Identified peptides | Identified proteins |
| --- | --- | --- | --- | --- | --- |
| K_DCM | 4 | 282639 | 25606 | 23874 | 13014 |
| K_100 | 4 | 268373 | 14235 | 17082 | 10773 |
| K_200 | 4 | 213489 | 4618 | 6852 | 6391 |
| K_500 | 4 | 158912 | 2799 | 5865 | 5520 |
| B1_DCM | 4 | 250238 | 10095 | 13408 | 9772 |
| B1_100 | 4 | 185664 | 3485 | 7488 | 6382 |
| B1_200 | 4 | 132075 | 2556 | 3166 | 2061 |
| B1_500 | 4 | 155469 | 2822 | 3131 | 1978 |
| B9_DCM | 4 | 237785 | 6971 | 11354 | 9339 |
| B9_100 | 4 | 200032 | 10020 | 12069 | 8996 |
| B9_200 | 4 | 180386 | 7053 | 9382 | 7563 |
| B9_500 | 4 | 176675 | 2619 | 4864 | 4330 |

**Supplementary Table 4.** The ratio of transporters in all proteins.

| **Transporter** | **All** | **Bacteria** | | **Eukaryota** | | **Archaea** | |
| --- | --- | --- | --- | --- | --- | --- | --- |
| **K2** |  |  |  |  |  |  |  |
| **K2_75** | 16.85 | 16.27 | 96.55% | 0.21 | 1.22% | 0.09 | 0.55% |
| **K2_DCM** | 11.07 | 9.18 | 82.90% | 1.41 | 12.75% | 0.35 | 3.13% |
| **K2_200** | 30.55 | 29.27 | 95.82% | 0.38 | 1.25% | 0.61 | 2.01% |
| **K2_500** | 14.06 | 13.72 | 97.60% | 0.11 | 0.75% | 0.09 | 0.66% |
| **B1** |  |  |  |  |  |  |  |
| **B1_DCM** | 11.83 | 9.93 | 83.95% | 1.44 | 12.20% | 0.14 | 1.17% |
| **B1_100** | 22.27 | 21.28 | 95.55% | 0.56 | 2.52% | 0.21 | 0.94% |
| **B1_200** | 6.89 | 6.68 | 97.00% | 0.10 | 1.42% | 0.05 | 0.68% |
| **B1_500** | 12.55 | 12.01 | 95.73% | 0.10 | 0.84% | 0.01 | 0.12% |
| **B9** |  |  |  |  |  |  |  |
| **B9_DCM** | 10.16 | 8.82 | 86.78% | 1.14 | 11.24% | 0.06 | 0.63% |
| **B9_100** | 19.54 | 17.32 | 88.66% | 1.72 | 8.81% | 0.24 | 1.23% |
| **B9_200** | 14.73 | 14.30 | 97.04% | 0.10 | 0.67% | 0.18 | 1.20% |
| **B9_500** | 14.20 | 13.84 | 97.46% | 0.22 | 1.54% | 0.03 | 0.24% |

# Supplementary Figures





**Supplementary Figure 1.** The location of the sampling sites in the Northwest Pacific Ocean. At the time of sampling, this region was influenced by the Kuroshio and Oyashio currents. Influenced by the Kuroshio Current, station K2 is a typical high temperature and low nutrient station. On the contrary, influenced by the Oyashio Current, B1 is a typical low temperature and high nutrient station. B9 is located between sites K2 and B1 and is influenced by the Kuroshio and Oyashio currents.


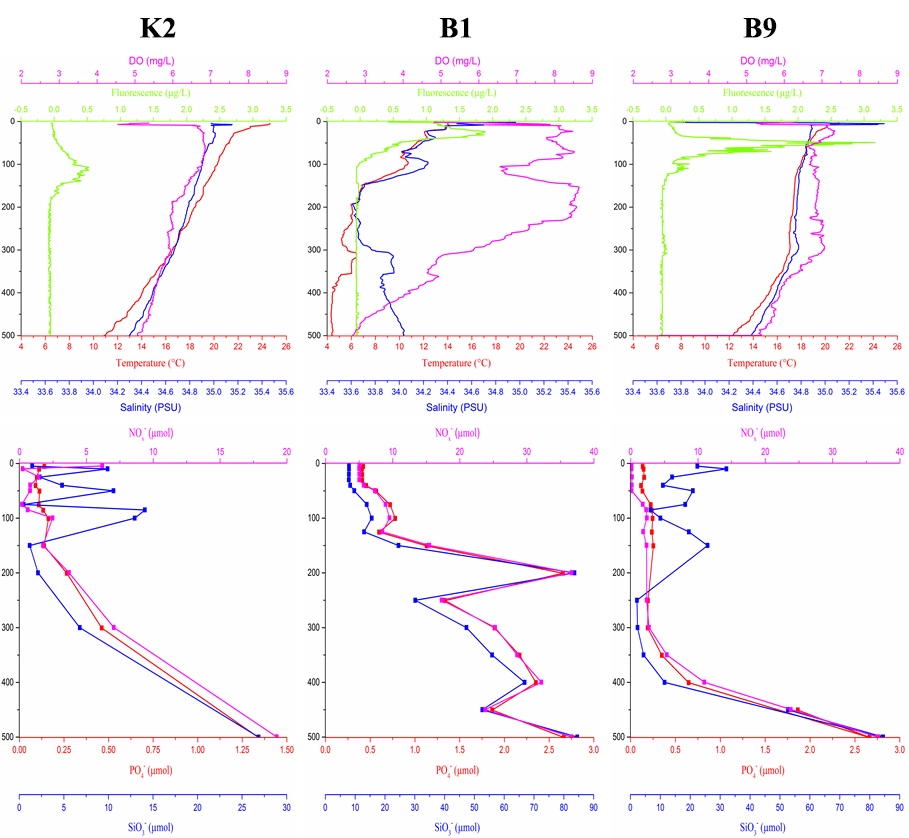


**Supplementary Figure 2.** Distribution of environmental parameters of three sampling sites in the northwest Pacific Ocean.


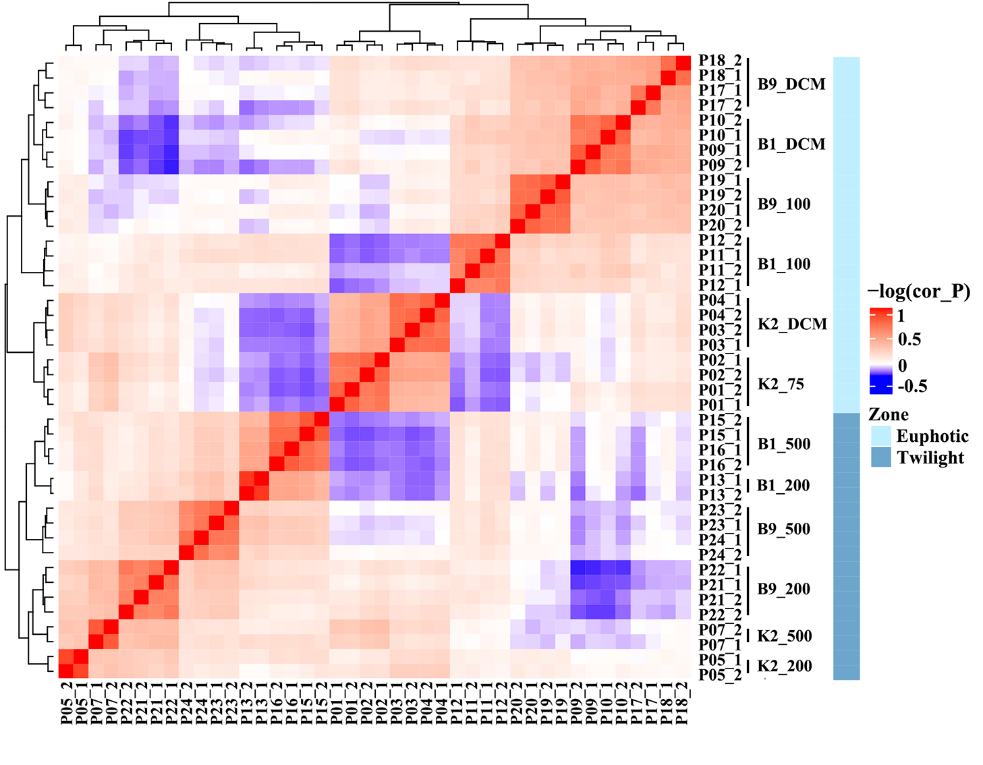


**Supplementary Figure 3.** Quality of metaproteomic data.





**Supplementary Figure 4.** Venn diagrams illustrating the relationship of protein similarity among the three sites.


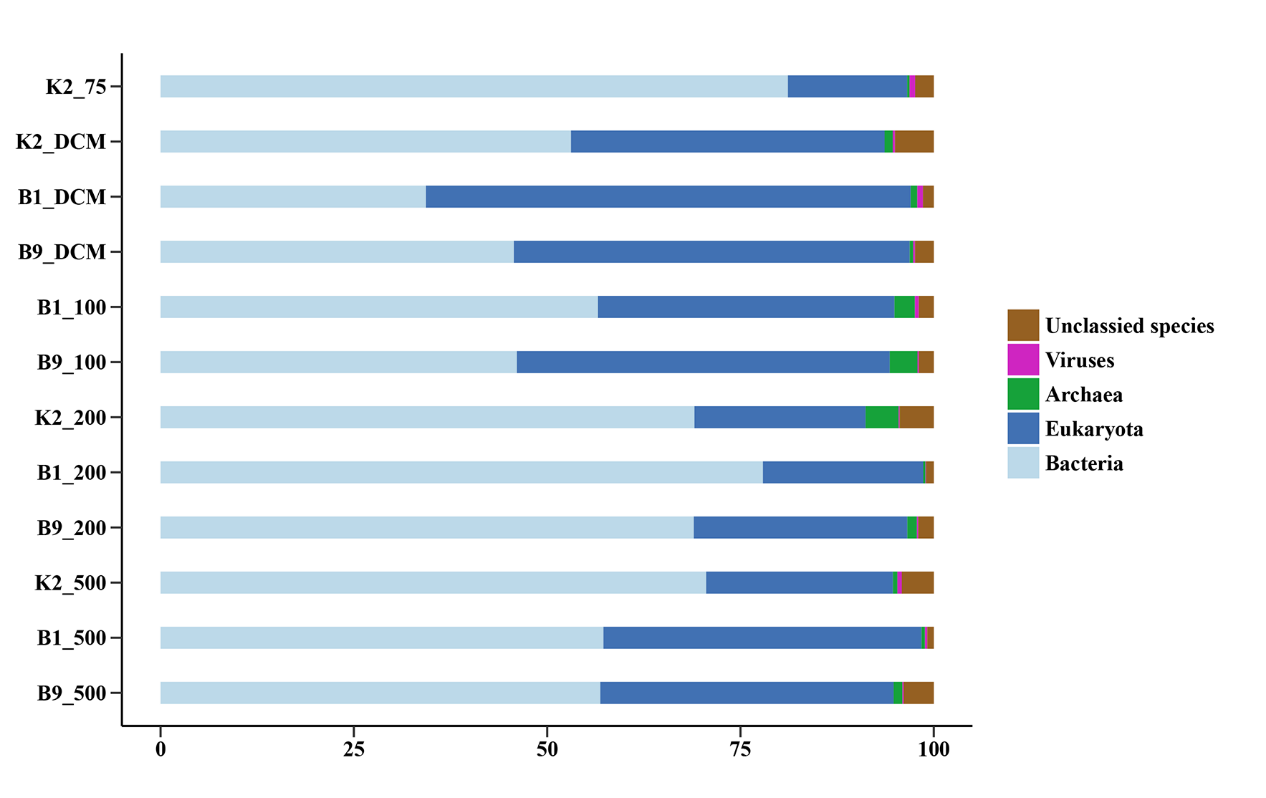


**Supplementary Figure 5** The distribution of the detected proteins among the three domains of life and viruses. Each detected protein was used to query NCBI-nr, using BLASTP to determine its closest relative. Sequence matches with bit scores less than 50 were considered unknown.


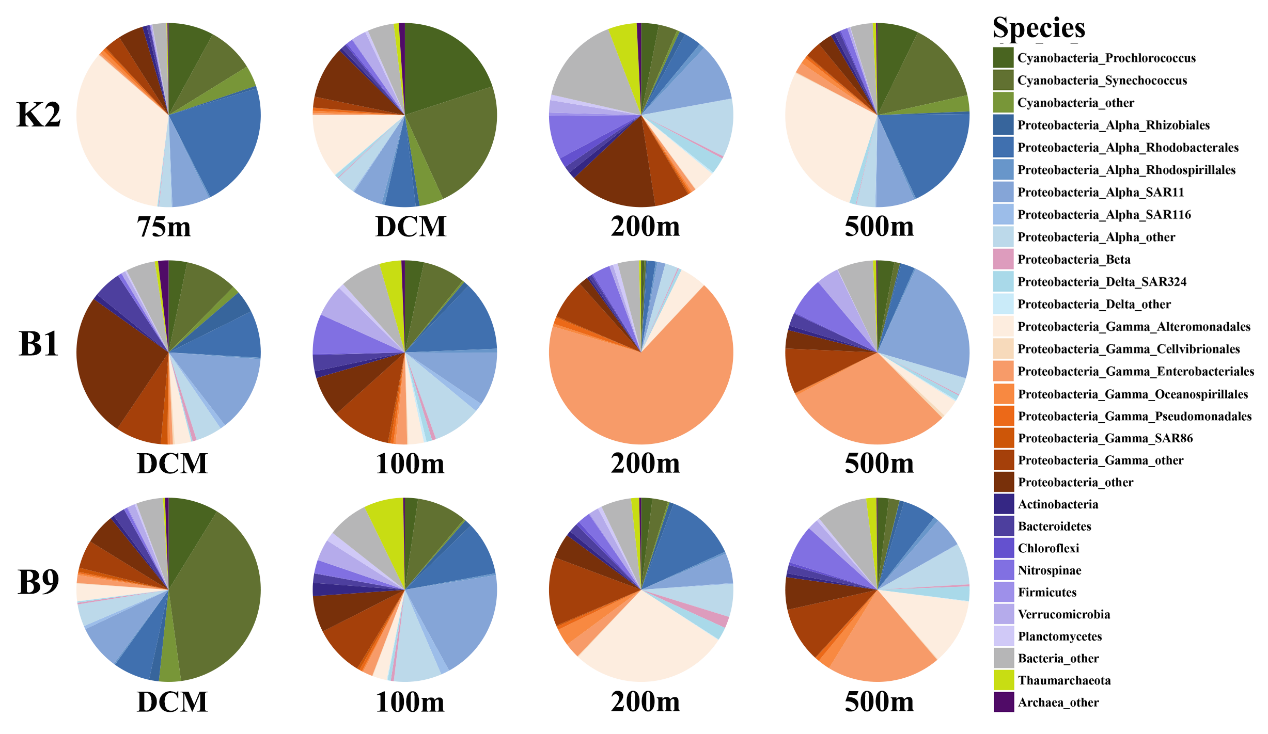


**(B)**

**(A)**


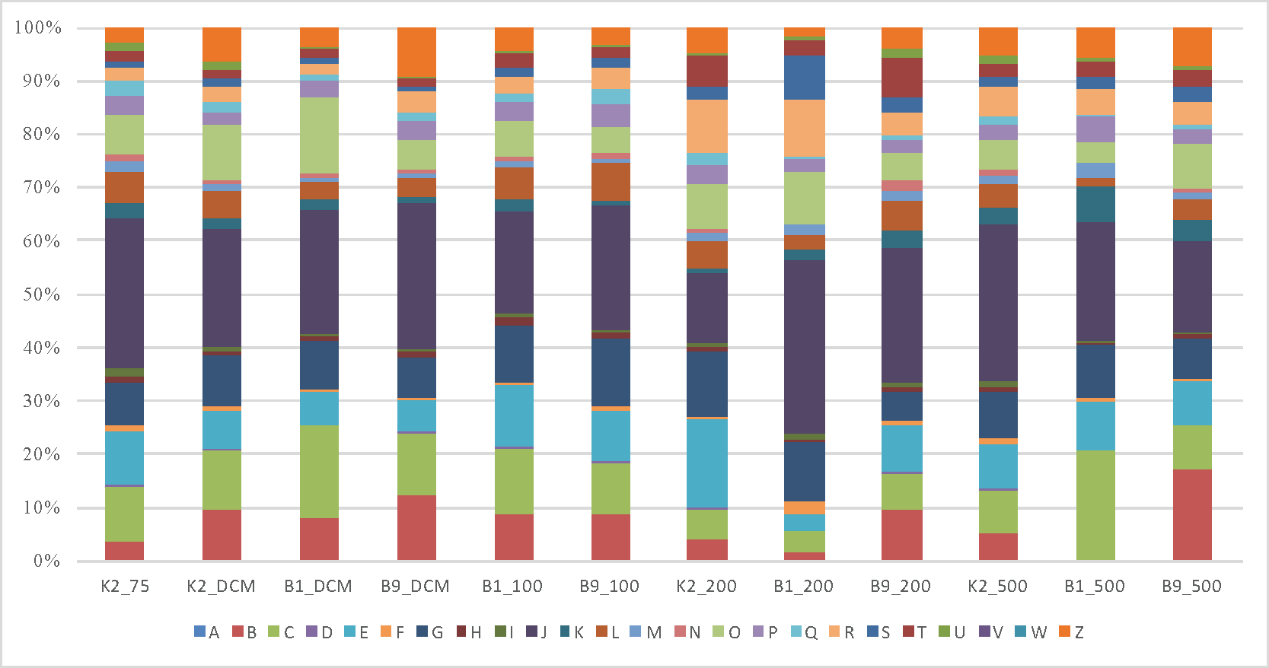


**Supplementary Figure 6.** (**A**) Taxonomic composition of bacterial communities identified by metaproteomics. Bacterial taxa with >1% relative abundance on average are displayed and named in the format phylum_class_order. (**B**) Abundance and distribution of functional classifications were inferred using COGs across sampling sites.
